# Palladium Hydroxide (Pearlman’s Catalyst) Doped MXene (Ti_3_C_2_Tx) Composite Modified Electrode for Selective Detection of Nicotine in Human Sweat

**DOI:** 10.3390/bios13010054

**Published:** 2022-12-29

**Authors:** Vasanth Magesh, Ashok K. Sundramoorthy, Dhanraj Ganapathy, Raji Atchudan, Sandeep Arya, Razan A. Alshgari, Ahmed Muteb Aljuwayid

**Affiliations:** 1 Centre for Nano-Biosensors, Department of Prosthodontics, Saveetha Dental College and Hospitals, Saveetha Institute of Medical and Technical Sciences, Poonamallee High Road, Velappanchavadi, Chennai 600077, Tamil Nadu, India; 2 School of Chemical Engineering, Yeungnam University, Gyeongsan 38541, Republic of Korea; 3 Department of Physics, University of Jammu, Jammu 180006, Jammu and Kashmir, India; 4 Department of Chemistry, College of Science, King Saud University, Riyadh 11451, Saudi Arabia

**Keywords:** MXene, palladium, electrochemical sensor, nicotine detection, human sweat, amperometry, sweat monitoring

## Abstract

High concentrations of nicotine (40 to 60 mg) are more dangerous for adults who weigh about 70 kg. Herein, we developed an electrochemical transducer using an MXene (Ti_3_C_2_Tx)/palladium hydroxide-supported carbon (Pearlman’s catalyst) composite (MXene/Pd(OH)_2_/C) for the identification of nicotine levels in human sweat. Firstly, the MXene was doped with Pd(OH)_2_/C (PHC) by mechanical grinding followed by an ultrasonication process to obtain the MXene/PHC composite. Secondly, XRD, Raman, FE-SEM, EDS and E-mapping analysis were utilized to confirm the successful formation of MXene/PHC composite. Using MXene/PHC composite dispersion, an MXene/PHC composite-modified glassy carbon electrode (MXene/PHC/GCE) was prepared, which showed high sensitivity as well as selectivity towards nicotine (300 µM NIC) oxidation in 0.1 M phosphate buffer (pH = 7.4) by cyclic voltammetry (CV) and amperometry. The MXene/PHC/GCE had reduced the over potential of nicotine oxidation (about 200 mV) and also enhanced the oxidation peak current (8.9 µA) compared to bare/GCE (2.1 µA) and MXene/GCE (5.5 µA). Moreover, the optimized experimental condition was used for the quantification of NIC from 0.25 µM to 37.5 µM. The limit of detection (LOD) and sensitivity were 27 nM and 0.286 µA µM^−1^ cm^2^, respectively. The MXene/PHC/GCE was also tested in the presence of Na^+^, Mg^2+^, Ca^2+^, hydrogen peroxide, acetic acid, ascorbic acid, dopamine and glucose. These molecules were not interfered during NIC analysis, which indicated the good selectivity of the MXene/PHC/GCE sensor. In addition, electrochemical determination of NIC was successfully carried out in the human sweat samples collected from a tobacco smoker. The recovery percentage of NIC in the sweat sample was 97%. Finally, we concluded that the MXene/PHC composite-based sensor can be prepared for the accurate determination of NIC with high sensitivity, selectivity and stability in human sweat samples.

## 1. Introduction

Nicotine (NIC) is one of the most addictive and hazardous drugs ever. As a main alkaloid, NIC [3-(1-methyl-2-pyrrolidinyl) pyridine] is found in tobacco leaves (*Nicotiana tabacum* L., Solanaceae) [1]. Initially, it was used for medical applications, but later, it became well known for its use in cigarettes. Overdose of NIC may lead to an increase of blood circulation to the heart, pulse rate, hypertension and artery contraction (blood carrying vessels). Nicotine may raise the risk of heart stroke as well as contribute to arterial wall hardening. This drug can stay in the body for up to 8 h based on how habitually it is used [2,3]. A fatal dose of NIC for kids is 10 mg (maximum), while for adults, it is 40 to 60 mg (0.5 to 1.0 mg kg^−1^ of body weight) [4]. Every day, more than a billion people use various forms of tobacco products, which causes them to become addicted to NIC. Nicotine is a tobacco-specific substance, which makes up of 95% of the entire alkaloid content in cigarettes and other tobacco products [5]. As a result, it is present in the human body fluids (0.5 mM to 0.8 mM of NIC in human saliva is considered a medically hazardous level) of both active and passive smokers (persons who had long term exposure to second-hand smoke) [6]. NIC is a substance that has an impact on the brain by evoking a sensation of euphoria, reviving energy and enhancing the mood, but it also poses some serious health risks, the most serious of which are heart, brain and respiratory conditions, causing lung carcinoma [7]. The World Health Organization (WHO) reported that tobacco usage is one of the leading causes of illness, as well as death, in India (causing around 1.35 million deaths each year). According to the CDCP (Centre for Disease Control and Prevention), smoking either passively or actively caused close to 0.16 million American deaths (affected by oral and lung cancer) in 2019 [8]. Apart from its negative impacts, research has been done to determine the therapeutic benefits of NIC for various diseases, such as ulcerative colitis, Alzheimer’s and Parkinson’s [7]. Thus, it is important to accurately determine NIC concentration in forensic samples, medications, human body fluids, toxicological samples, etc. Generally, NIC determination was performed by the use of high-performance liquid chromatography (HPLC) [9,10], chemiluminescence [11], capillary electrophoresis with mass spectrometry (CE/MS) [12], HPLC with mass spectrometry (HPLC/MS) [13], immunoassays [14], gas chromatography [15,16] and other chromatographic techniques [15,16,17,18]. These methods have some limitations, such as the requirement of a pretreatment process for each sample, the complex instrumentation system needed, the requirement of trained technicians and the high cost.

Recently, electrochemical sensors have received more attention in the development of NIC sensors because of their portability, simplicity, faster analysis time, high sensitivity and low cost [19,20]. Additionally, amperometry (AMP), square wave voltammetry (SWV) and differential pulse voltammetry (DPV) techniques were used for the determination of NIC with the lowest limit of detection (LOD) [21]. NIC analysis can be carried out using a bare GCE, but it showed an oxidation peak at highly positive potential, which is the outside of the potential range of most working electrodes (e.g., glassy carbon electrode) [22]. Additionally, other interference compounds (oxidation peaks) could prevent the NIC from being determined selectively [6]. Thus, it is necessary to adjust the sensitivity and selectivity of the sensor by using nanomaterials that are able to oxidize NIC at lower potential. As a result, the modified electrodes could improve the LOD, dynamic range, analyte adsorption, sensitivity and selectivity. There have been significant developments in electrochemical (bio-) sensors based on nanostructured materials over the past several years [23,24,25,26]. Specifically, 2D nanomaterials such as graphene [27,28,29] and MoS_2_ [30,31] have undergone substantial research, both individually and as a part of nanocomposite structure for the creation of responsive sensing platforms. However, 2D nanomaterials have also exhibited some limitations, such as poor electrical conductivity (MoS_2_), excessive hydrophobicity (MoS_2_, graphene) and inert surfaces [32,33]. In graphene, only the surface flaws and edges can be functionalized, and functionalization of MoS_2_ was also considerably more challenging [34]. The large-scale production of these kind of materials is also a labor-intensive process that further inhibits advancement in this area.

MXene, which was discovered in 2011 by Yuri Gogotsi, has greater electrical conductivity, as well as high hydrophilicity, as a result of surface termination groups (-F, =O, and -OH), better ion exchange behavior, convenient surface functionalization and credible bulk production, making it the perfect material for the development of high efficiency electrochemical biosensors [35]. MXene was obtained from the MAX phase by removing ‘A’ atom layers, where ‘M’ is an early transition metal, ‘A’ is a 13/14 group-element, and ‘X’ can be N or C [36]. There are more than 200 distinct stable MXene phases as projected based on the combinations of several transition metals, such Ti, Mo, V, Cr and their mixtures with N and C [37]. The unique properties of MXenes include their great electrical conductivity, good hydrophilicity, strong chemical stability, large surface area, surface tunability, possible synthesis in bulk volumes, ecofriendly traits and harmlessness [38]. MXene exhibits structural and electrical properties similar to those of other 2D materials such as graphene, metal oxides and so on. MXene has the clear benefit of high electrical conductivity, which is essential for enhancing the process of heterogeneous electron transport. One of the crucial reasons for its use in electrochemical sensors is its outstanding electroconductive feature [39]. The 2D-layered structure and distinctive surface with numerous chemical groups allowed MXene to be coupled with a wide range of functional elements or biomolecules for various analytical applications [40]. Ti_3_C_2_ is the most common type of MXene, which is the ideal choice for the fabrication of electrochemical biosensors.

The MXene’s surface was functionalized to enhance the sensitivity and selectivity for the determination of a particular analyte. Palladium nanoparticles (Pd NPs) are the most extensively studied among the noble metals and metal oxide nanoparticles due to their superior electrical properties and catalytic activity [41]. Furthermore, palladium may interact and strongly bind to the 2D materials (graphene) because of the development of more transmission channels and interaction states between them [42]. As a result of the oxygenated functional groups on its surface, Pd NPs can be easily functionalized on 2D materials, and can accelerate catalytic activity.

In this study, we reported an electrochemical sensor made of MXene/PHC composite for the determination of the nicotine level in a sweat sample without any pretreatment process. The mechanochemical synthesis approach was used to synthesize the MXene/PHC composite. PHC doping might have improved the stability of MXene dispersion via functional groups on the surface. Further, Raman, FE-SEM, EDX, E-mapping and XRD characterizations were carried out to confirm the formation of the MXene/PHC composite. The PHC-doped MXene (Ti_3_C_2_T_x_)-modified GCE was characterized by electrochemical measurements and also used to determine the NIC concentration by amperometry analysis. In a phosphate buffer solution (pH = 7.4), MXene/PHC/GCE showed remarkable electrocatalytic activity towards NIC oxidation at a lower potential compared to MXene/GCE and bare GCE. Interestingly, MXene/PHC/GCE exhibited a linear response for NIC, from 0.25 µM to 37.5 µM (Sensitivity = 0.328 µA µM^−1^ cm^2^), with an LOD of 27 nM. Further studies on selectivity, repeatability and stability were carried out, which indicated that the MXene/PHC sensor can be used for the determination of NIC with excellent selectivity and stability, even in the presence of other interfering substances. Finally, the MXene/PHC-based sensor was utilized to quantify the amount of nicotine present in a human sweat sample.

## 2. Experimental

### 2.1. Chemicals and Materials

Potassium ferrocyanide (K_4_Fe(CN)_6_), potassium ferricyanide (K_3_Fe(CN)_6_), potassium chloride (KCl), magnesium chloride (MgCl_2_), glacial acetic acid and dopamine were purchased from Sisco Research Laboratories (SRL) Pvt. Ltd., Mumbai, India. Sodium chloride (NaCl), palladium hydroxide (Pd(OH)_2_)-supported carbon (PHC) and nicotine (3-(1-methyl-2-pyrrolidinyl) pyridine) were obtained from Sigma-Aldrich, Bengaluru, India. Aluminum metal powder (~300 mesh) and titanium metal powder (~352 mesh) were acquired from Alfa Aesar, Mumbai, India. Sodium dihydrogen phosphate monohydrate (H_2_NaPO_4_·H_2_O) and sodium phosphate dibasic heptahydrate (Na_2_HPO_4_·7H_2_O) were supplied by Spectrochem Pvt. Ltd., Mumbai, India. Nafion was received from Sainergy Fuel Cell India Pvt. Ltd., Chennai, India, Vitamin C (L-ascorbic acid) was obtained from Hi-Media Laboratories. Calcium chloride (CaCl_2_) was purchased from SD Fine-Chem Ltd., Mumbai, India.

### 2.2. Characterizations

A field-emission scanning electron microscope (FE-SEM, JSM IT800 (JEOL, Tokyo, Japan)) was used to examine the surface morphology of the MXene and MXene/PHC composite. XPLORE-30 (High Wycombe, Oxford, UK) was used to capture energy dispersive X-ray spectra (EDS) and elemental mapping (E-map) images. A 532 nm laser excitation was used to acquire all Raman spectra (XPLORA plus (Horiba, Kyoto, Japan)). X-ray diffraction (XRD) data was collected by the D8-Advance XRD instrument (BRUKER, Billerica, MS, USA). HPLC experiments were carried out using an i-series LC-2050C 3D (SHIMADZU, Kyoto, Japan) and methanol-ammonium acetate mobile phase was used. A pH meter (LH-10—(Lab Holic, Chennai, India)) fitted with the combination electrode (reference glass electrode) was used to measure all pH readings. All electrochemical studies, including electrochemical impedance spectroscopy (EIS), amperometry (i-t curve) and cyclic voltammetry (CV) were carried out on a CHI 760E workstation (CH Instruments, Austin, TX, USA) with a three-electrode configuration. The electrochemical cell contains a reference electrode (Ag/AgCl soaked in 3 M KCl), a working electrode (GCE with a 0.07 cm^2^ working area) and a counter electrode (Pt wire).

### 2.3. Preparation of MAX Phase (Ti_3_AlC_2_) and MXene (Ti_3_C_2_T_x_)

The MAX phase was prepared as reported elsewhere (Ti_3_AlC_2_) [43]. In a nutshell, the stoichiometric ratio of 3:1:2 of titanium, aluminum and graphite precursors were combined and mechanically mixed well in a ball-mill for 360 min. After that, a high temperature split-tube furnace with inert environment (N_2_) was used to anneal the milled mixture for 120 min at 1100 °C. Finally, Ti_3_AlC_2_ (MAX powder) was collected. Later, the Al layer was selectively etched out to obtain MXene powder. For this purpose, 1 g of MAX powder was treated with hydrofluoric acid (HF 48%) for 24 h at room temperature. Furthermore, to produce MXene powder, the resultant dispersion was diluted with distilled water and centrifuged (at 5000× *g* rpm for 10 min and repeated this process for 18–20 times) to neutralize the pH of the supernatant. Finally, the obtained MXene precipitate was dried at 100 °C in a hot air-oven for few days.

### 2.4. Preparation of MXene/PHC Composite

**Figure 1:**
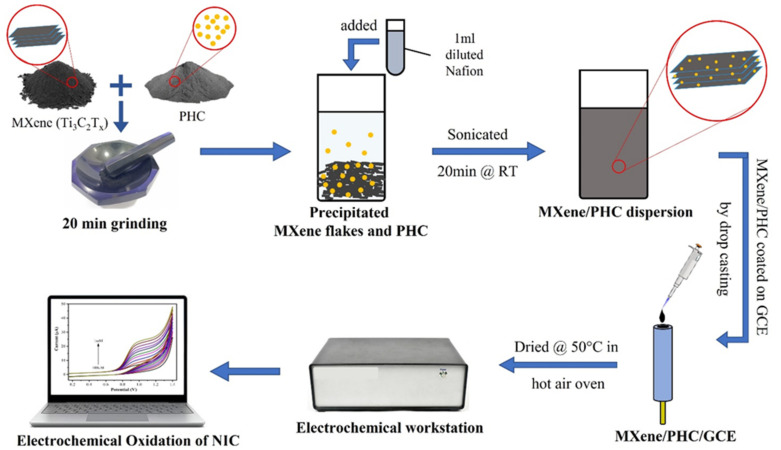
Schematic illustration of MXene/PHC composite synthesis for the sensor development to determine NIC concentration.

To develop the MXene/PHC composite, MXene powder (5 mg) and palladium hydroxide-supported carbon (1:1 ratio) (Pearlman’s catalyst) (5 mg) were mixed and grinded with the help of a mortar and pestle for 20 min to make fine particle composite. This mixture was transferred to a suitable container and ultrasonicated for 20 min at RT with 1 mL of diluted nafion (Nf) (a mixture of 1 mL distilled water (DI H_2_O) + 1 mL ethanol + 10 µL nafion (5 wt.%)), as shown in Figure 1. The MXene/PHC composite dispersion was again diluted with DI water at a 1:50 ratio (composite: DI water).

### 2.5. Fabrication of MXene/PHC Sensor

To begin, the glassy carbon electrode (GCE of 3 mm diameter) was delicately cleaned using the standard washing process to achieve a reflective surface [43]. Next, 8 µL of the MXene/PHC dispersion was drop-casted over the GCE surface and dried in a hot air oven at 50 °C (Figure 1). After that, MXene/PHC/GCE was slightly immersed in DI water to remove any loosely bounded particles from the modified electrode surface. In addition, the MXene/GCE (without PHC) was prepared in the same way for the examination of the samples to confirm that MXene/PHC is superior to MXene for NIC sensing.

### 2.6. Electrochemical Measurements

The GCE was first polished with 0.05 micron alumina powder to create a reflecting surface, and cyclic voltammetry (CV) was then performed in a solution of 0.1 M KCl with 5 mM [Fe(CN)_6_]^4−/3−^ using bare GCE in the potential range of −0.2 to 0.6 V. The CV was then utilized to investigate the electrochemical behavior of NIC on bare GCE in the potential window of 0 V to +1.2 V in 0.1 M phosphate buffer at a scan rate of 50 mV s^−1^ (Figure 1). The voltametric responses of bare GCE, MXene/GCE, and MXene/PHC/GCE were studied in the same potential window with various concentrations of nicotine in 0.1 M phosphate buffer (pH = 7.4). The pH studies were performed in different pH buffer solutions with the addition of the same concentration (1 mM) of nicotine. The phosphate buffer (from pH 6–8), KCl/HCl mixture (pH 2), acetic acid/sodium acetate buffer (pH 4) and NH_4_Cl/ammonia solution (pH 11) were utilized in this experiment as a buffer solution with 0.1 M concentration. The linear range of nicotine determination (0.25 µM–37.5 μM) was performed by an amperometry (i-t curve) analysis. The calibration plot was made using oxidation currents and NIC concentrations (amperograms were recorded for three times (n = 3) and the average value with standard deviation are provided). The recovery studies were performed by amperometry to determine the nicotine content in real-world and standard samples, respectively.

### 2.7. Real Sample Collection and Extraction

The sweat sample was collected from a smoker (who smokes 5–7 cigarettes per day, Age = 30). The participant was instructed to conduct strenuous physical workouts for around 2 h. The segregated sweat was then wiped with a pure thin cotton cloth, then squeezed and transferred to a 15 mL centrifuge tube. After that, the collected sweat was centrifuged for 10 min at 10,000× *g* rpm to separate the supernatant, then it was stored at 4 °C for further examination (Figure S1 in Supplementary Materials).

## 3. Results and Discussion

### 3.1. Material Characterizations

#### 3.1.1. Raman and XRD Analysis

**Figure 2:**
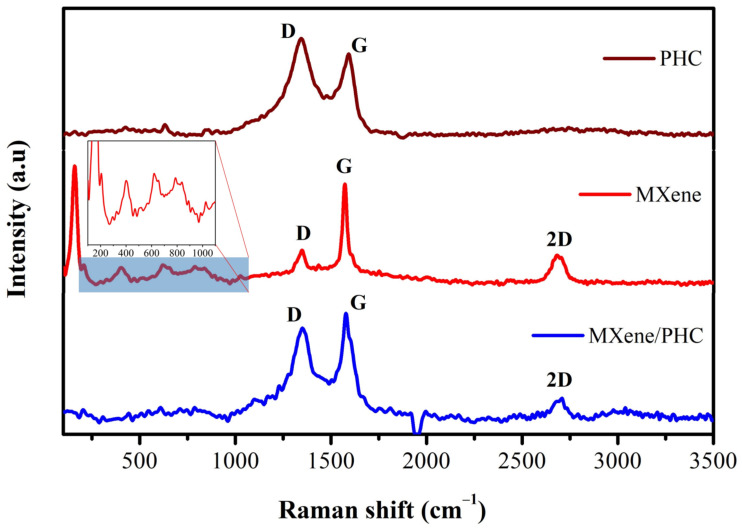
Raman spectra of PHC, MXene and MXene/PHC composite. Inset: an enlarged spectrum of MXene.

The Raman spectra of PHC, MXene (Ti_3_C_2_T_x_) and MXene/PHC were successfully recorded. The palladium hydroxide dry basic carbon (Figure 2, brown curve) showed two major peaks of D and G bands at ~1343 cm^−1^ and ~1591 cm^−1^, respectively. The MXene (Figure 2, red curve) displayed a broad band at around 158 cm^−1^, which corresponded to the Eg (doubly degenerative vibration state) of TiO [44]. Furthermore, three low intensity bands at 399 cm^−1^, 618 cm^−1^ and 799 cm^−1^ were found due to the vibrations of MXene atoms [45,46]. In addition, three more Raman bands were found at ~1350 cm^−1^, ~1572 cm^−1^ and ~2689 cm^−1^, which correspond to the D, G and 2D bands of graphitic carbon (Figure 2). The D-band represents the defects of graphitic carbon and the G-band represents the vibrations of sp^2^ hybridized atoms of carbon in a 2D-hexagonal lattice [47]. The I_D_/I_G_ peak intensity ratios of the Raman spectra were used to quantify the degree of disorder [48]. The I_D_/I_G_ ratio (0.86) was increased for the MXene/PHC composite compared to MXene (0.37), which indicated the existence of PHC in the composite. Furthermore, the Raman spectrum of the MXene/PHC composite (Figure 2, blue curve) showed the existence of MXene/PHC-specific bands (D, G as well as 2D bands).

Compared to the MXene spectrum, the D, G, and 2D bands of MXene/PHC were found to be down shifted (blue shifted) and detected at lower spectral range (~9 cm^−1^). In addition, compared to MXene, the D-band intensity was increased (from 73 to 117) and the G-band intensity was decreased (from 197 to 136) compared to the MXene/PHC composite. This study revealed that PHC might have interacted with the functional groups of MXenes and also confirmed the composite formation.

**Figure 3:**
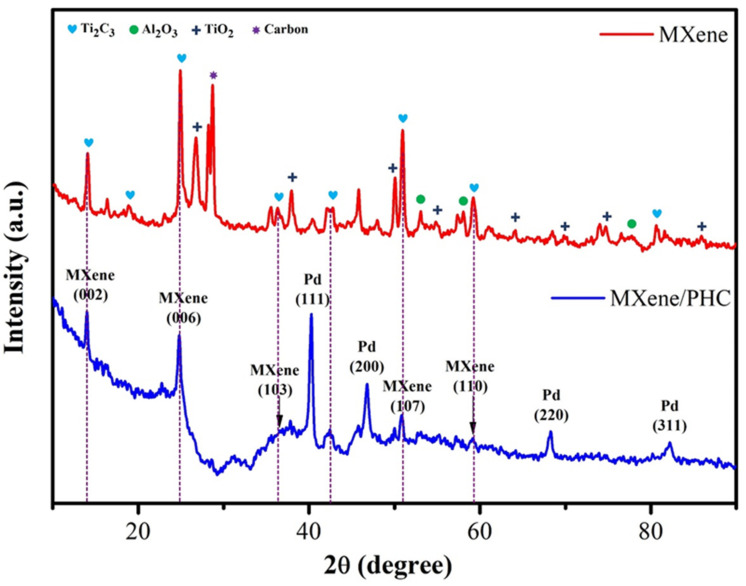
XRD spectra of MXene and MXene/PHC composite.

The crystalline properties of MXene and MXene/PHC composite were determined by XRD analysis (Figure 3). The diffraction peaks (2θ value) of MXene observed at 14.57° (002), 18.96° (004), 24.8° (006), 36.41° (103), 50.79° (107) and 59.22° (110) [49,50], which are matched with JCPDS No. 52-087 and confirmed the MXene formation. In addition, some XRD bands were observed at 2θ values of 26.7°, 37.94°, 49.9°, 54.7°, 64.1°, 69.7° and 74.7°, and 53.1°, 58.1° and 77.8°, which corresponded to the oxide peaks of TiO_2_ and Al_2_O_3_, respectively. Additionally, the graphitic carbon peak was found at 28.6° (red curve). From this XRD spectrum, the synthesis of mixed phase MXene was confirmed [43,51]. Moreover, the XRD pattern of MXene/PHC composite was encompassed with all of the distinct peaks of MXene (14.57°, 24.8°, 36.41°, 50.79° and 59.22°) and PHC (40.2°, 46.7°, 68.3° and 82.1°), as shown in Figure 3 (blue curve). The 2θ values of PHC were well matched with the previously reported research articles [52,53].

This data further confirmed the successful synthesis of the MXene/PHC composite. Furthermore, after the composite formation, some of the MXene peak intensities decreased and a minor peak shift was noticed. These changes could be the result of the integration of PHC into MXene as well as variations in the lattice parameters in the composite crystal structure [54].

#### 3.1.2. FE-SEM, EDS and E-Mapping Analysis

**Figure 4:**
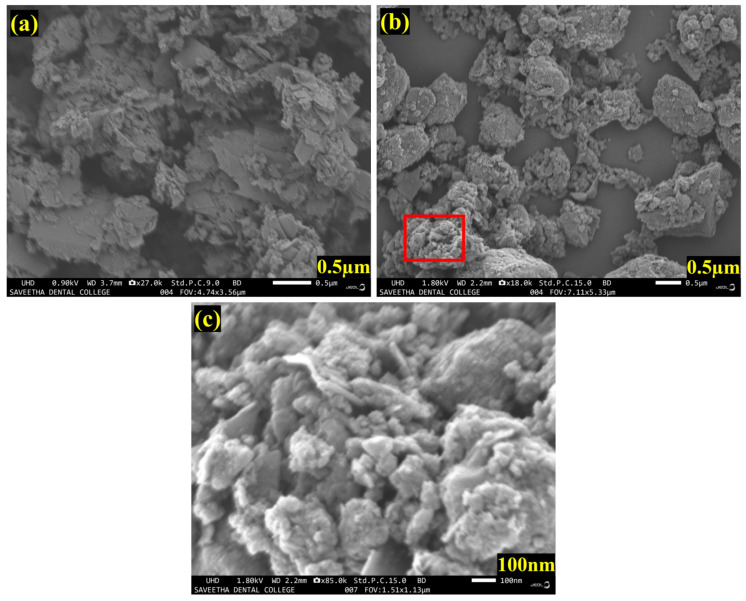
FE-SEM images of (**a**) stacked layer of MXene sheets, (**b**) MXene/PHC composite and (**c**) enlarged image of (**b**), as highlighted in the red square.

As shown in Figure 4a, FE-SEM images of as-synthesized mixed phase MXene (Ti_3_C_2_T_x_) revealed a stacked layered structure. Our synthesis process clearly produced a few-layered MXene with a thickness of 100–400 nm. It can be seen that the FE-SEM image of the MXene/PHC composite showed the successful incorporation of PHC within the MXene layers (Figure 4b,c).

**Figure 5:**
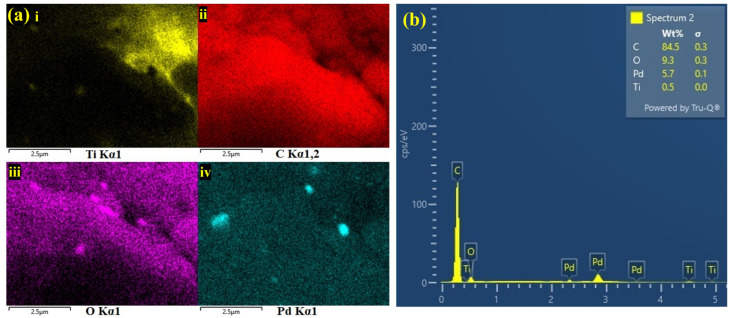
(**a**) Elemental mappings were performed on MXene/PHC composite: (**i**) titanium, (**ii**) carbon, (**iii**) oxygen and (**iv**) palladium. (**b**) EDS spectrum of MXene/PHC composite.

Figure 4c is the highlighted part of Figure 4b with 100 nm resolution, which confirmed the composite of MXene flakes and PHC. The FE-SEM images validated that the PHC was evenly decorated on the MXene’s surface. Furthermore, energy dispersive spectroscopy (EDS) revealed the presences of titanium (Ti), carbon (C), Pd and oxygen (O), which again confirmed the effective formation of the MXene/PHC composite. The elemental mapping (E-mapping) of the samples also revealed the homogenous distribution of all elements in the MXene/PHC composite, as shown in Figure 5a. The existence of corresponding elements (in atomic percentages) in the MXene/PHC composite were also recorded by EDS analysis (Figure 5b).

### 3.2. Electrochemical Properties of MXene/PHC Composite

**Figure 6:**
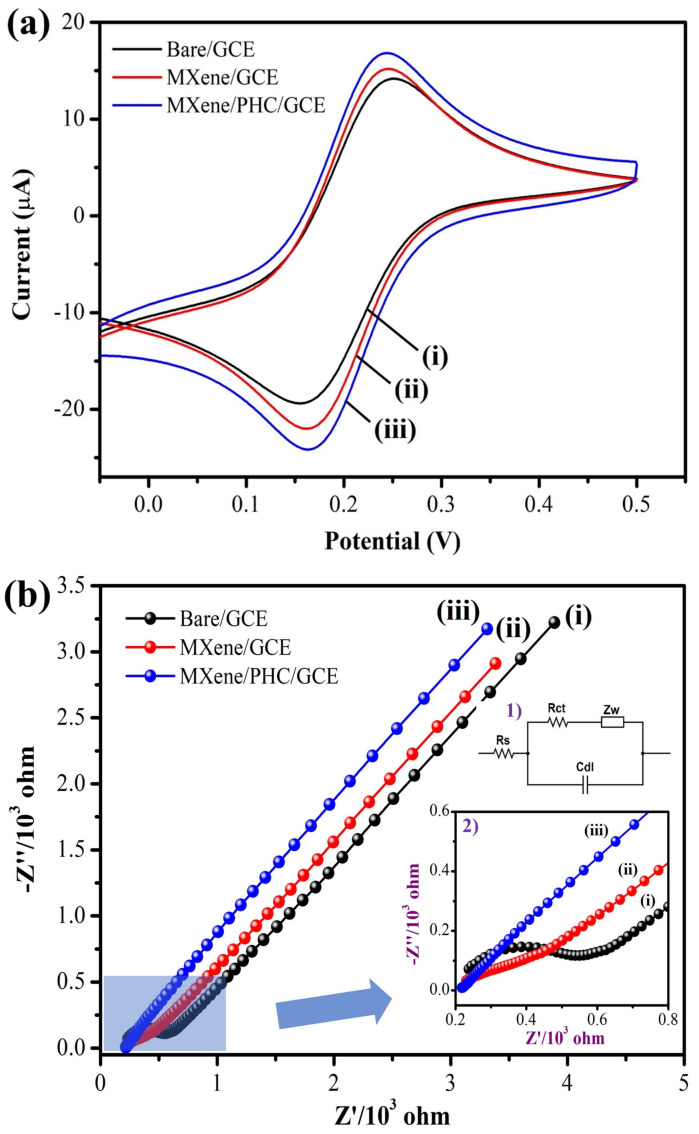
(**a**) CVs and (**b**) EIS spectra of (**i**) bare GCE, (**ii**) MXene/GCE and (**iii**) MXene/PHC/GCE in 0.1 M KCl with 2 mM [Fe(CN)_6_]^3−^. Inset: (**1**) Randle’s circuit and (**2**) enlarged area of highlighted part of the spectrum.

The electrochemical properties of the MXene/PHC composite were investigated by using CV in 0.1 M phosphate buffer and EIS in 0.1 M KCl with 2 mM [Fe(CN)_6_]^3−^. Initially, the CV was recorded with a bare GCE in 2 mM of [Fe(CN)_6_]^3−^ solution, which yielded anodic (E_pa_) and cathodic (E_pc_) peak potentials at 0.251 V and 0.152 V, respectively (Figure 6a, black curve). The oxidation and reduction peak potentials of [Fe(CN)_6_]^3−^ on the MXene/GCE were located at +0.246 and +0.161 V. For MXene/PHC/GCE, E_pa_ and E_pc_ of [Fe(CN)_6_]^3−^ were found at +0.243 and +0.164 V, respectively. Moreover, MXene/PHC/GCE had increased (11.1% against MXene/GCE and 22.7% against bare GCE) the I_pa_ and I_pc_ of [Fe(CN)_6_]^3−^ redox peak compared to MXene/GCE and bare/GCE, respectively (Figure 6a, red and blue curves), which indicated the high conductivity and enhanced surface area of MXene/PHC/GCE. The Nyquist plots were recorded for the bare/GCE (i), MXene/GCE (ii) and MXene/PHC/GCE’s (iii) to scrutinize the interfacial electron transfer resistance (R_ct_) of fabricated sensors [55] (Figure 6b). The Rcts of each electrode were calculated from the Nyquist plots using the semicircle’s diameter, which was located in the high-frequency region. Among these, MXene/PHC/GCE (0.01 Ω) showed lower Rct compared to MXene/GCE (115.3 Ω) and bare/GCE (283.5 Ω). The absence of a semicircle in the case of MXene/PHC/GCE indicated that the charge transfer resistance was substantially reduced because of the good conductivity of the electrode [56]. EIS indicated that MXene/PHC/GCE possesses excellent conductivity after the incorporation of PHC with MXene. Moreover, the MXene/PHC/GCE-modified electrode showed a higher electron transfer efficacy due to its possession of the lowest Rct value.

#### 3.2.1. Electrochemical Oxidation of NIC

**Figure 7:**
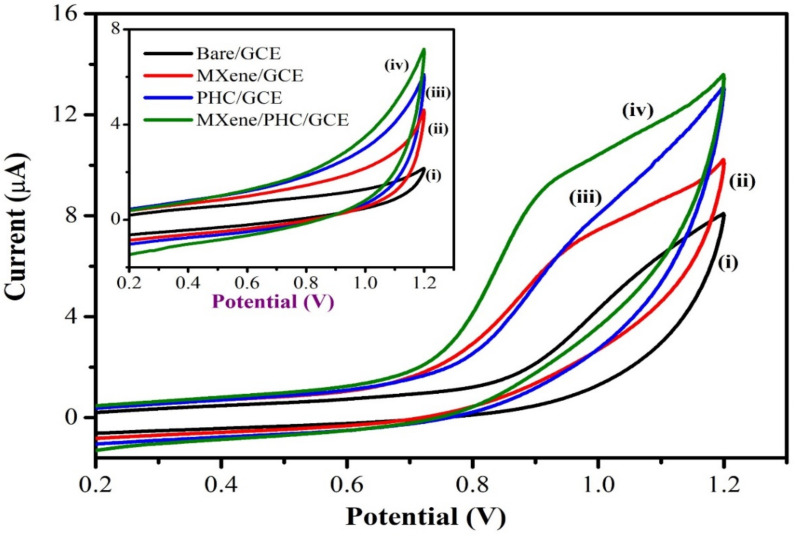
CVs were recorded in 0.1 M phosphate buffer (pH 7.4) with 0.3 mM of NIC using (**i**) bare/GCE, (**ii**) MXene/GCE, (**iii**) PHC/GCE and (**iv**) MXene/PHC/GCE at a scan rate of 50 mV s^−1^. Inset: CVs of each electrode in 0.1 M phosphate buffer (pH 7.4) without NIC.

The cyclic voltammograms (CVs) were recorded using bare/GCE, MXene/GCE, PHC/GCE and MXene/PHC/GCE in 0.1 M phosphate buffer before and after the addition of 300 µM of NIC. Before addition of NIC, the MXene/PHC/GCE exhibited CV curve with a higher background current than MXene/GCE, PHC/GCE and bare GCE (Figure 7 (inset)). There were no oxidation or reduction peaks observed in the potential window of +0.2 V to +1.2 V. However, when NIC (300 µM) was added, the MXene/PHC/GCE exhibited a higher oxidation peak current (8.9 µA) at +0.9 V than MXene/GCE (5.5 µA), PHC/GCE (5.3 µA) and bare GCE (2.1 µA) (Figure 7). According to earlier reports, NIC begins to oxidize at a potential of ~0.9 V on the modified GCE [7,29,36,57].

**Figure 8:**
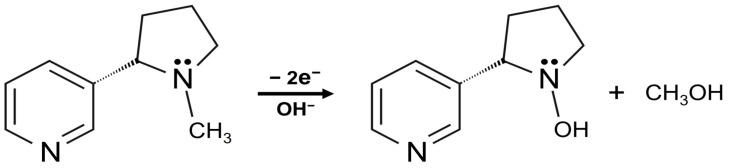
Schematic representation of oxidation mechanism of the nicotine.

Figure 8 shows the electro-oxidation mechanism of NIC on MXene/PHC/GCE [58]. During a cathodic scan, no reduction peak was observed until +0.2 V. As a result, NIC oxidation is an irreversible process at MXene/PHC/GCE, which is also corroborated by previous reports [30,31]. It was clear that the NIC oxidation peak was noticed at a lower potential on the composite modified electrode (+0.9 V) compared to bare GCE (+1.1 V) (Figure 7 curves i–iv). In addition, the MXene/PHC/GCE exhibited the highest oxidation peak current for NIC compared to MXene/GCE and PHC/GCE, which indicated that the MXene/PHC composite had exhibited a greater catalytic effect towards NIC.

#### 3.2.2. Detecting NIC Concentration

**Figure 9:**
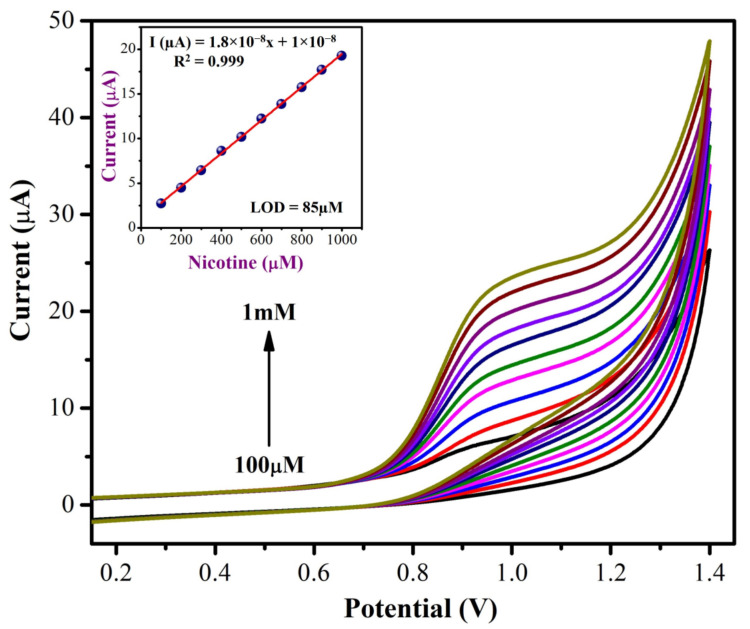
CVs were recorded with different concentrations of NIC (from 100 µM to 1 mM) using MXene/PHC/GCE in 0.1 M phosphate buffer (pH 7.4) at a scan rate of 50 mV s^−1^. Inset: Calibration curve of NIC, which was plotted between different NIC concentrations (µM) and I_pa_ of NIC (µA).

**Figure 10:**
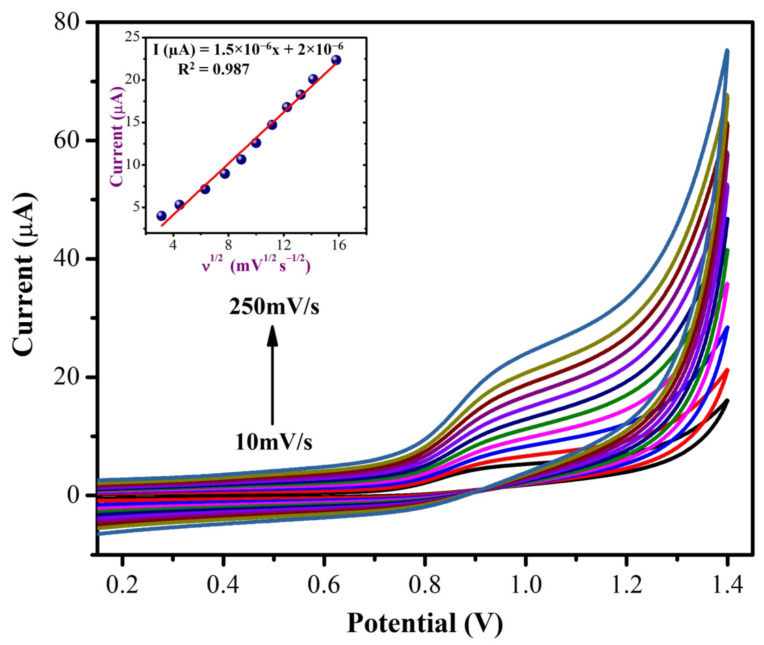
Different scan rate studies were carried out using a MXene/PHC composite modified GCE with 0.5 mM NIC. Inset: A linear curve was made between the square root of scan rate (mV s^−1^) vs. oxidation current (µA).

Using CVs, the concentrations of NIC were determined using MXene/PHC/GCE in 0.1 M phosphate buffer at a scan rate of 0.05 V s^−1^. The concentrations of NIC varied from 100 µM to 1 mM. As shown in Figure 9, the oxidation peak currents (I_pa_) of NIC were increased linearly with the concentrations. This demonstrated the high sensitivity and stability of the MXene/PHC/GCE towards the NIC oxidation reaction. Next, to study the effect of scan rates on NIC oxidation, CVs were recorded with the varied scan rates from 10 mV s^−1^ to 250 mV s^−1^ in 0.1 M phosphate buffer with 500 µM NIC using MXene/PHC/GCE. The oxidation peak currents (I_pa_) of NIC were gradually increased with respect to the square root of the scan rate, as shown in Figure 10. According to the kinetic controlled process, the oxidation peak potential (E_pa_) of NIC slowly moved to the positive side. The linear equation was found to be Y = 1.5 × 10^−6^x + 2 × 10^−6^ with a correlation coefficient (R^2^) of 0.987 (Figure 10, Inset). This data confirmed that a diffusion-controlled oxidation process of NIC occurred on the MXene/PHC/GCE [59,60]. In order to find out the suitable pH for NIC analysis, CVs were recorded at different pH buffers containing 1 mM nicotine (from pH 2 to 11) using a MXene/PHC/GCE sample.

**Figure 11:**
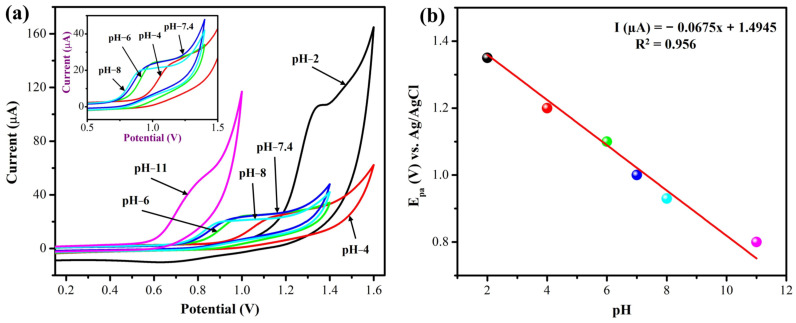
(**a**) CVs were carried out in different pH buffer solutions with 1 mM NIC at a scan rate of 50 mV s^−1^. Inset: Enlarged view of CVs of pH 4, 6, 7.4 and 8. (**b**) Relationship between the different pH and oxidation potential of NIC. (The CVs were recorded in different buffer solutions using the 1 mM nicotine. The phosphate buffer (pH 6–8), KCl/HCl buffer (pH 2), acetic acid/sodium acetate buffer (pH 4) and NH_4_Cl/ammonia solution (pH 11) were utilized in this experiment with 0.1 M concentration).

The I_pa_ and E_pa_ of nicotine were examined in different pH buffer solutions (pH 2–11), as shown in Figure 11a, b. It was observed that the oxidation peak potential of NIC started to move towards the negative potential when the pH of the buffer increased; it demonstrated that the electro-oxidation of NIC involves the same number of protons. Simultaneously, the I_pa_ of NIC was higher in pH 2 and 11, while in the other pH 4 range, almost the same current responses were observed. If we look more closely, the I_pa_ of NIC was higher in pH 7.4 than in pH 6. By considering the pH of human body fluids (pH 5.5 to pH 8), we adopted pH 7.4 to determine the NIC in human sweat. Additionally, Figure 11b displayed the relationship between the pH and E_pa_ of NIC, which gave a slope of −67.5 mV/pH, a value that was similar to the theoretical value (−59 mV/pH) of the Nernst equation. This demonstrated that the nicotine oxidation process on the MXene/PHC/GCE was involved with the same number of electrons and protons [61].

#### 3.2.3. Amperometry and Interference Studies

**Figure 12:**
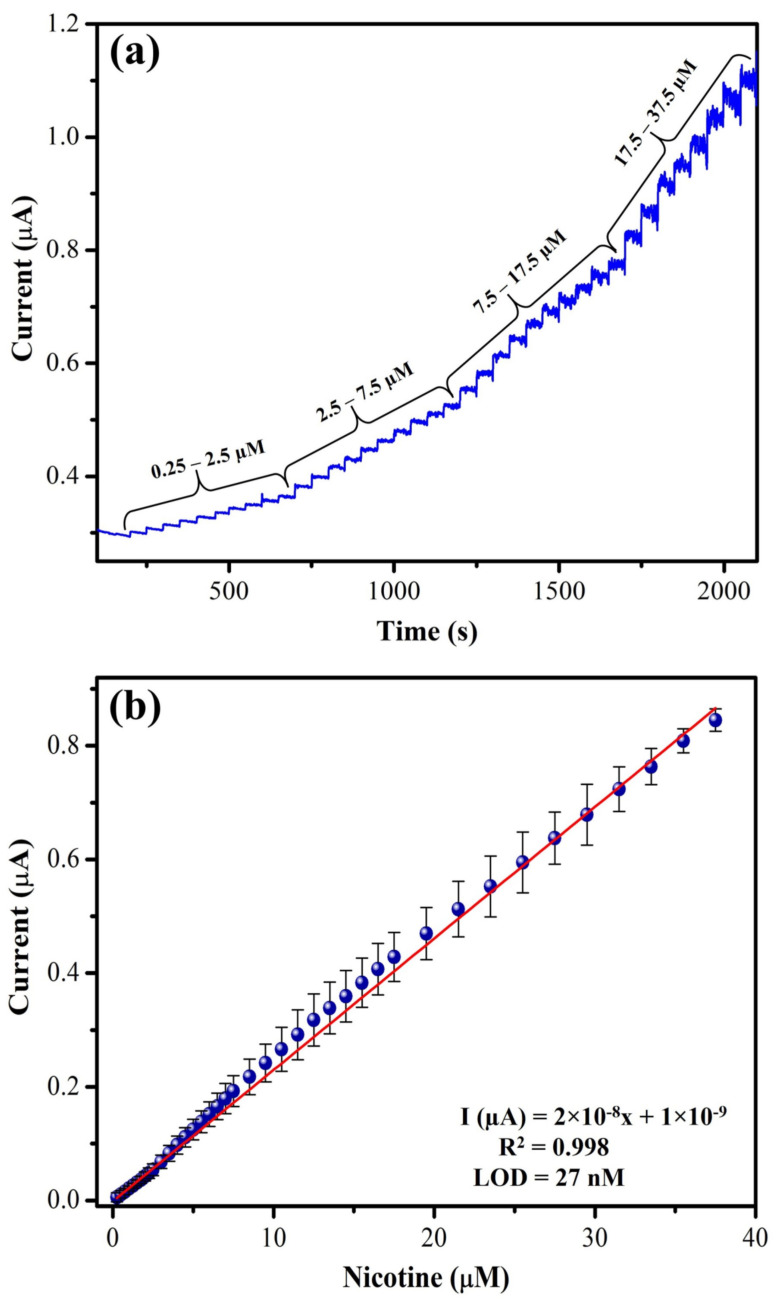
Amperometry (i-t curve) performance of MXene/PHC/GCE (**a**) for linear determination of various concentrations of NIC (from 0.25 µM to 37.5 µM) in 0.1 M phosphate buffer at an applied potential of 1 V. (**b**) Calibration graph of NIC was made between different concentrations (µM) vs. current response (µA). During the analysis, phosphate buffer was stirred with a magnetic stirrer at 750 rpm. The symbol and error bars represented the mean and SD (standard deviation) values (n = 3), respectively.

The amperometry technique was carried out to investigate the linear range NIC determination at +1.0 V using a MXene/PHC/GCE in 0.1 M phosphate buffer. When NIC concentrations were increased from 0.25 µM to 37.5 µM [28,57,62], MXene/PHC/GCE produced a linear current response for each addition of nicotine by amperometry (Figure 12), and the sensitivity was calculated as 0.286 µA µM^−1^ cm^2^. In the dynamic linear range of determination, amperometry is more accurate than CV. The obtained high sensitivity of the sensor could be attributed to the quick response by electrocatalytic oxidation of NIC by MXene/PHC/GCE [63]. The LOD of nicotine was measured as 27 nM. It should be mentioned that at the higher concentration of NIC, the steady state current began to drop because the catalyst’s active regions of the modified electrode might have been saturated. The analytical performance of the proposed method is also compared with other NIC sensors. Table 1 shows the comparison of the linear range of detection, LOD and oxidation potential of NIC. It can be mentioned that our sensor’s performance is relatively comparable to other reported methods.

**Figure 13:**
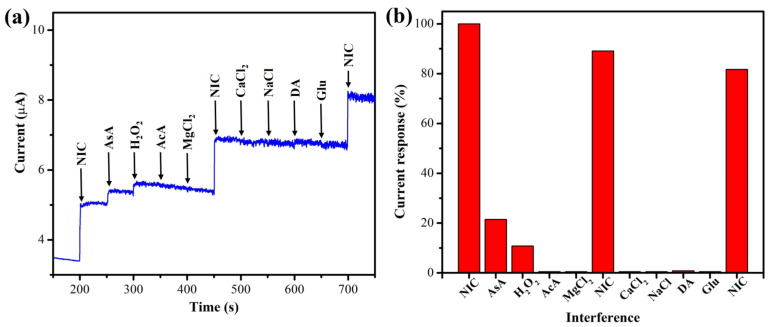
(**a**) Interference analysis was carried out in the presence of (5 µM) NIC in 0.1 M phosphate buffer (pH 7.4) at 1 V using a MXene/PHC/GCE (rotation rate = 750 rpm). (**b**) The bar graph shows the percentage of current response after each addition of interfering compounds along with NIC (5 µM).

To ascertain the selectivity of newly developed MXene/PHC/GCE, amperogram (i-t) was recorded with the additions of interference compounds and NIC (5 µM), as shown in Figure 13a. The commonly present interfering chemicals in sweat and their oxidation responses were examined (examples: ascorbic acid (AsA), dopamine (DA, 0.5 µM), and glucose (Glu), acetic acid (AcA), magnesium chloride (MgCl_2_), calcium chloride (CaCl_2_), sodium chloride (NaCl) and hydrogen peroxide (H_2_O_2_)). All interference compounds were added to 0.1 M phosphate buffer (pH 7.4) with NIC at a 1:1 ratio. It can be noted that the oxidation current of NIC was reduced by about 10% when these chemicals were coexisted with NIC, as shown in Figure 13b. Thus, it was confirmed that these studied compounds did not strongly interfere with the determination of nicotine.

#### 3.2.4. Repeatability and Stability Studies

**Figure 14:**
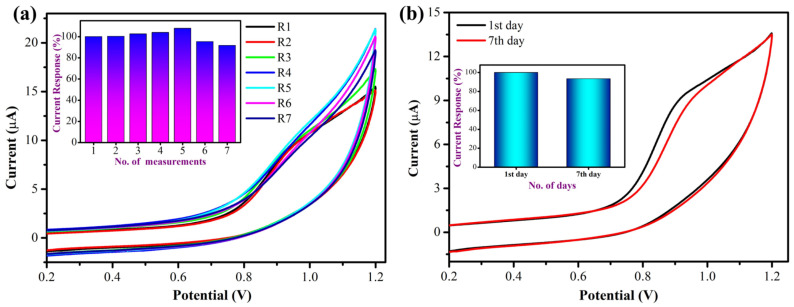
(**a**) Repeatability measurements of 300 µM NIC using a same MXene/PHC/GCE up to seven repeated measurements in 0.1 M phosphate buffer (Inset shows the bar graphs of No. of measurements vs. NIC oxidation peak current response of each measurement). (**b**) The stability of the MXene/PHC/GCE towards the oxidation of 300 µM NIC in 0.1 M phosphate buffer (Inset: bar diagram shows current responses of NIC vs. no. of days).

The newly prepared MXene/PHC/GCE was used to investigate the oxidation of 300 µM NIC in 0.1 M phosphate buffer in order to evaluate the sensor’s repeatability and analyze the precision of the modified electrode (Figure 14a). The differences in the oxidation peak current (I_pa_) of NIC at +0.95 V are clearly shown in the inset of (Figure 14a). The I_pa_ of NIC was slightly increased at +0.95 V during the first five independent measurements of NIC. Following that, the I_pa_ of NIC started to decrease after the sixth and seventh consecutive measurements, possibly due to material leaching out of the GCE after the fifth measurement. These results showed that the MXene/PHC composite has reasonably good stability for multiple repeated experiments.

Likewise, the stability of the MXene/PHC/GCE was investigated by oxidation of NIC in 0.1 M phosphate buffer (pH = 7.4) from the first to the seventh days (Figure 14b). For this study, the MXene/PHC/GCE was newly prepared and used to measure the oxidation current of 300 µM NIC in 0.1M phosphate buffer. After this measurement, the MXene/PHC dispersion was stored at room temperature. After 7 days of storage, the MXene/PHC dispersion was used to prepare the sensor, which showed a well-defined oxidation peak for 300 μM of NIC (inset Figure 14b). However, the oxidation peak current was decreased about 7% compared to its initial electro-catalytic activity. This study indicated that the MXene/PHC dispersion was relatively stable and could be used for the preparation of stable sensors for nicotine analysis.

#### 3.2.5. Real-World Sample Analysis

**Figure 15:**
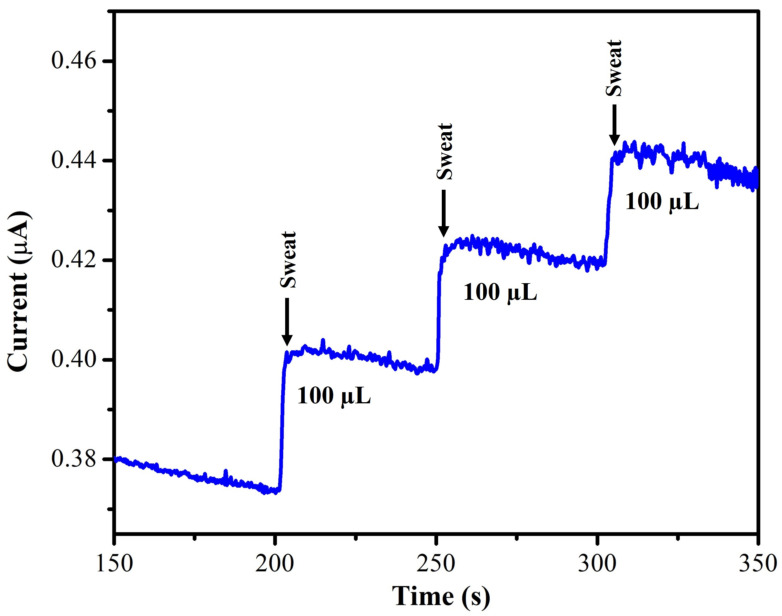
NIC determination in real-world sample (sweat) by amperometry at 1 V in 0.1 M phosphate buffer (pH 7.4) with a rotation rate of 750 rpm.

A human sweat sample was collected and evaluated in order to determine the effectiveness of the MXene/PHC composite-based sensor for real-world applications. All samples were examined in triplicate measurements (n = 3) by amperometry to determine the nicotine level, and the results are displayed as the average of the three measurements. The collected sweat (supernatant) sample was diluted (sweat and DI water with 1:2 ratio) and added to the 0.1 M phosphate buffer (pH = 7.4), where the designated sensor recorded the oxidation current response. The current response was increased after each addition of a sweat sample (Figure 15).

**Figure 16:**
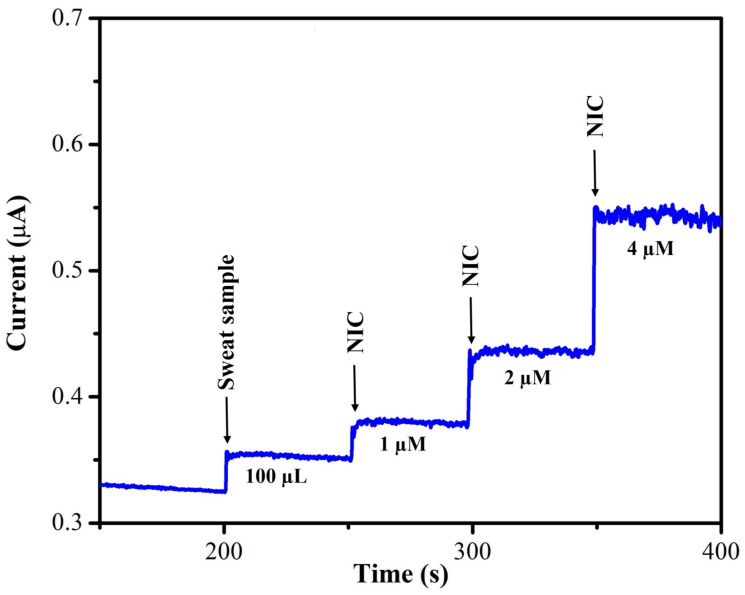
Amperogram was recorded to calculate the recovery percentage by using MXene/PHC/GCE in sweat sample and various concentrations of standard nicotine (NIC) solutions added in to 0.1 M phosphate buffer. PBS was stirred with rotation rate of 750 rpm at RT.

Moreover, high-performance liquid chromatography (HPLC) was used to identify nicotine in the real sample to ensure that the results produced by the MXene/PHC/GCE sensors were accurate (Figure S2). The recovery studies for NIC were carried out on the human sweat sample (Figure 16). Initially, 100 µL of sweat was added to the 0.1 M phosphate buffer (pH = 7.4), after that, NIC was spiked in to the solution mixture (sweat + phosphate buffer), followed by the addition of three different concentrations of NIC (1 µM, 2 µM, and 4 µM). The amperogram was recorded using the MXene/PHC/GCE at +1.0 V and the calibration curve was used to calculate the unknown concentration of NIC in the sweat sample. Table 2 depicts the estimated nicotine concentration in the human sweat sample by using amperometry and also spiked standard NIC concentrations, which demonstrated the good recovery of spiked NIC in the sweat sample (97–103%).

## 4. Conclusions

A MXene/PHC composite-based electrochemical sensor was used for the first time to determine the nicotine concentration in a human sweat sample with good specificity and sensitivity. The developed composite was comprehensively characterized by FE-SEM, EDS, E-mapping, Raman and XRD, which confirmed that the MXene/PHC composite was successfully formed with a uniform distribution of elements. Next, the MXene/PHC/GCE-based electrochemical sensor was prepared for NIC oxidation. MXene/PHC/GCE enhanced the oxidation peak current of nicotine compared to MXene/GCE and bare/GCE. It was found that NIC was oxidized electrocatalytically on the MXene/PHC composite by involving an equal quantity of protons and electrons. Thus, the newly developed MXene/PHC composite could be used to develop an effective sensor for NIC determination in various samples. In addition, MXene/PHC/GCE provided a wide linear range of NIC determination, from 0.25 µM to 37.5 µM, with LOD of 27 nM, as well as capable of sensing nicotine at physiological condition compared to other studies (Table 1). In addition, the nicotine concentration in human sweat sample was determined using the MXene/PHC-based sensor in 0.1 M phosphate buffer. Finally, we believe that the MXene/PHC-based sensor could be used to determine the NIC concentration in a human sweat sample with high selectivity.

## Figures and Tables

**Table 1:** Electrochemical detection of NIC using various electrochemical sensors and compared with our method.

Electrode	Catalyst	Electrolyte	E_pa_ (V)	Technique	Linear Range (µM)	LOD (µM)	Test Sample	Reference
SPCE	N-GrS	PB, pH = 7.4	0.85	CV	0–200	0.047	Cigarettes/Urine	[6]
GCE	CuWO_4_/rGO/Nf	PB, pH = 7	1.1	CV	0.1–0.9	0.035	Cigarettes/Urine	[7]
BDD	-	BRB, pH = 8	1.35	DPV	0.5–202.5	0.3	Tobacco products/Pharmaceuticals	[22]
GCE	HBN/Gr	PB, pH = 7.4	0.97	AMP	1–1000	0.42	Tobacco samples	[29]
SPCE	-	PB, pH = 7.5	0.9	DPV	1–375	0.6	Sweat	[57]
CPE	Ce-NPs	BRB, pH = 2	1	CV	4–500	0.0943	Cigarettes	[62]
GCE	TiO_2_/PEDOT	PB, pH = 7.4	0.88	AMP	0–5000	4.9	-	[64]
GCE	MWCNT/ACS	PB, pH = 8	0.65	AMP	5–1395	1.42	-	[65]
GCE	MWCNT	Na_2_C_2_O_4_, pH = 4.5	1.4	DPV	31–1900	9.3	Cigarettes	[66]
BPPGE	MWCNT	PB, pH = 8	0.65	CV	0–1000	1.5	-	[67]
CPE	TiO_2_	BRB, pH = 5	0.87	CV/DPV	2–540	0.0134	Cigarettes/Urine	[68]
GCE	MWCNT/NCB	PB, pH = 7.4	0.72	CV	10–50	5	-	[69]
SPE	CNC	PB, pH = 7	0.75	CV	10–1000	2	Artificial Saliva	[70]
GCE	P-AHNSA	PB, pH = 7.5	0.88	SWV	1–200	0.866	Cigarettes	[71]
PGE	SDS (surfactant)	PB, pH = 7	0.84	SWV	7.6–107.5	2	Cigarettes	[72]
A-GCE	–	PB, pH = 7	0.83	SWV	1–200	0.7	Cigarettes	[73]
GCE	MXene/PHC	PB, pH = 7.4	1	CV/AMP	0.25–37.5	0.027	Human sweat	Current work

Footnotes: **E_pa_**—oxidation or anodic peak potential, **I_pa_**—oxidation or anodic peak current, **LOD**—limit of detection, **BBD**—boron doped diamond electrode, **GCE**—glassy carbon electrode, **SPCE**—screen printed carbon electrode, **CPE**—carbon paste electrode, **BPPGE**—basal plane pyrolytic graphite electrode, **SPE**—screen printed electrode, **PGE**—pencil graphite electrode, **A-GCE**—activated glassy carbon electrode. **TiO_2_/PEDOT**—titanium dioxide/poly(3,4-ethylenedioxythiophene), **CuWO_4_/rGO/Nf**—copper tungstate/reduced graphene oxide/nafion, **MWCNT/ACS**—multiwalled carbon nanotube/alumina-coated silica, **N-GrS**—nitrogen doped graphene sheets, **Ce-NPs**—cerium nanoparticles, **MWCNT/NCB**—multiwalled carbon nanotube/nano carbon black, **CNC**—carbon nanotube cluster, **P-AHNSA**—Poly (4-Amino-3-Hydroxynaphthalene Sulfonic Acid), **SDC**—anionic surfactants, **HBN/Gr**—hexagonal boron nitride/graphene. **BRB**—Britton-Robinson buffer solution, **PB**—phosphate buffer solution, **Na_2_C_2_O_4_**—sodium oxalate buffer. **CV**—cyclic voltammetry, **DPV**—differential pulse voltammetry, **AMP**—amperometry, **SWV**—square wave voltammetry.

**Table 2:** Determination of NIC concentration in human sweat sample using MXene/PHC/GCE sensor.

S.No.	Samples	Added (µM)	Found * (µM)	Recovery (%)
1.	Human Sweat	-	0.91	-
2.	Std. NIC	1.0	1.88	97.0
3.	Std. NIC	2.0	2.89	99.0
4.	Std. NIC	4.0	5.05	103.5

* For the std. NIC, the found value was subtracted by NIC concentration of sweat before recovery calculation.

## Data Availability

Data can be made available upon request.

## Supplementary Materials

The following supporting information can be downloaded at: https://www.mdpi.com/article/10.3390/bios13010054/s1, Figure S1: Schematic illustration of sweat sample collection and electrochemical analysis of nicotine; Figure S2: HPLC spectra of standard nicotine (NIC) solution and real sweat sample were recorded. Reference [74] is cited in Supplementary Materials.

## References

[1] Nouri F., Nourollahi-Fard S.R., Foroodi H.R., Sharifi H. (2016). In Vitro Anthelmintic Effect of Tobacco (Nicotiana Tabacum) Extract on Parasitic Nematode, Marshallagia Marshalli. J. Parasit. Dis. Off. Organ Indian Soc. Parasitol..

[2] Benowitz N.L., Burbank A.D. (2016). Cardiovascular Toxicity of Nicotine: Implications for Electronic Cigarette Use. Trends Cardiovasc. Med..

[3] US Department of Health and Human Services (2010). How Tobacco Smoke Causes Disease. The Biology and Behavioral Basis for Smoking-Attributable Disease: A Report of the Surgeon General.

[4] Goldstein A.L., Faulkner B., Wekerle C. (2013). The Relationship among Internal Resilience, Smoking, Alcohol Use, and Depression Symptoms in Emerging Adults Transitioning out of Child Welfare. Child Abuse Negl..

[5] Bhartiya D., Kumar A., Kaur J., Kumari S., Sharma A.K., Sinha D.N., Singh H., Mehrotra R. (2018). In-Silico Study of Toxicokinetics and Disease Association of Chemicals Present in Smokeless Tobacco Products. Regul. Toxicol. Pharmacol..

[6] Li X., Zhao H., Shi L., Zhu X., Lan M., Zhang Q., Hugh Fan Z. (2017). Electrochemical Sensing of Nicotine Using Screen-Printed Carbon Electrodes Modified with Nitrogen-Doped Graphene Sheets. J. Electroanal. Chem..

[7] Karthika A., Karuppasamy P., Selvarajan S., Suganthi A., Rajarajan M. (2019). Electrochemical Sensing of Nicotine Using CuWO4 Decorated Reduced Graphene Oxide Immobilized Glassy Carbon Electrode. Ultrason. Sonochem..

[8] Siegel R.L., Miller K.D., Jemal A. (2019). Cancer Statistics, 2019. CA Cancer J. Clin..

[9] Kim J.-Y., Lee S.-M., Chang M.-I., Cho Y.-J., Lee H.-J., Chae Y.-S., Rhee G.-S. (2013). Development of a Method for Analyzing the Nicotine Content in Synthetic Flavoring Substances as Unauthorized E-Cigarette Liquid by Using HPLC. Korean J. Food Sci. Technol..

[10] Vindatiche I., Roche D., Callais F., Lequang N.T., Labrousse F. (2000). Analytical Improvements in Barlow Reaction Coupled to HPLC Detection of Nicotine and ITS Metabolites. J. Liq. Chromatogr. Relat. Technol..

[11] Ramírez N., Özel M.Z., Lewis A.C., Marcé R.M., Borrull F., Hamilton J.F. (2012). Determination of Nicotine and N-Nitrosamines in House Dust by Pressurized Liquid Extraction and Comprehensive Gas Chromatography—Nitrogen Chemiluminiscence Detection. J. Chromatogr. A.

[12] Baidoo E.E.K., Clench M.R., Smith R.F., Tetler L.W. (2003). Determination of Nicotine and Its Metabolites in Urine by Solid-Phase Extraction and Sample Stacking Capillary Electrophoresis-Mass Spectrometry. J. Chromatogr. B.

[13] Vieira-Brock P.L., Miller E.I., Nielsen S.M., Fleckenstein A.E., Wilkins D.G. (2011). Simultaneous Quantification of Nicotine and Metabolites in Rat Brain by Liquid Chromatography–Tandem Mass Spectrometry. J. Chromatogr. B.

[14] Langone J.J., Gjika H.B., Van Vunakis H. (1973). Nicotine and Its Metabolites. Radioimmunoassays for Nicotine and Cotinine. Biochemistry.

[15] Hossain A.M., Salehuddin S.M. (2013). Analytical Determination of Nicotine in Tobacco Leaves by Gas Chromatography–Mass Spectrometry. Arab. J. Chem..

[16] Shrivas K., Patel D.K. (2010). Liquid-Phase Microextraction Combined with Gas Chromatography Mass Spectrometry for Rapid Determination of Nicotine in One-Drop of Nightshades Vegetables and Commercial Food Products. Food Chem..

[17] Aragón M., Marcé R.M., Borrull F. (2013). Determination of N-Nitrosamines and Nicotine in Air Particulate Matter Samples by Pressurised Liquid Extraction and Gas Chromatography-Ion Trap Tandem Mass Spectrometry. Talanta.

[18] Kuhn J., Vollmer T., Martin C., Hendig D., Knabbe C. (2012). Fast and Sample Cleanup-Free Measurement of Nicotine and Cotinine by Stable Isotope Dilution Ultra-Performance Liquid Chromatography–Tandem Mass Spectrometry. J. Pharm. Biomed. Anal..

[19] Rodsud S., Limbut W. (2019). A Simple Electrochemical Sensor Based on Graphene Nanoplatelets Modified Glassy Carbon Electrode (GrNPs/GCE) for Highly Sensitive Detection of Yohimbine (YOH). J. Electrochem. Soc..

[20] Martins E.C., Santana E.R., Spinelli A. (2023). Nitrogen and Sulfur Co-Doped Graphene Quantum Dot-Modified Electrode for Monitoring of Multivitamins in Energy Drinks. Talanta.

[21] Inam O., Demir E., Uslu B. (2020). Voltammetric Pathways for the Analysis of Ophthalmic Drugs: A Review. Curr. Pharm. Anal..

[22] Švorc Ľ., Stanković D.M., Kalcher K. (2014). Boron-Doped Diamond Electrochemical Sensor for Sensitive Determination of Nicotine in Tobacco Products and Anti-Smoking Pharmaceuticals. Diam. Relat. Mater..

[23] Nagarajan R.D., Murugan P., Sundramoorthy A.K. (2020). Selective Electrochemical Sensing of NADH and NAD+ Using Graphene/Tungstate Nanocomposite Modified Electrode. ChemistrySelect.

[24] Kumar S.A., Chen S.-L., Chen S.-M. (2010). Electrochemical Sensing of H[Sub 2]O[Sub 2] at Flavin Adenine Dinucleotide/Chitosan/CNT Nanocomposite Modified Electrode. Electrochem. Solid-State Lett..

[25] Kalambate P.K., Li Y., Shen Y., Huang Y. (2019). Mesoporous Pd@ Pt Core–Shell Nanoparticles Supported on Multi-Walled Carbon Nanotubes as a Sensing Platform: Application in Simultaneous Electrochemical Detection of Anticancer Drugs Doxorubicin and Dasatinib. Anal. Methods.

[26] Huang X.-J., Choi Y.-K. (2007). Chemical Sensors Based on Nanostructured Materials. Sens. Actuators B Chem..

[27] Rajendran J., Kannan T.S., Dhanasekaran L.S., Murugan P., Atchudan R., ALOthman Z.A., Ouladsmane M., Sundramoorthy A.K. (2022). Preparation of 2D Graphene/MXene Nanocomposite for the Electrochemical Determination of Hazardous Bisphenol A in Plastic Products. Chemosphere.

[28] Rajendran J., Reshetilov A.N., Sundramoorthy A.K. (2021). An Electrochemically Exfoliated Graphene/Poly(3,4-Ethylenedioxythiophene) Nanocomposite-Based Electrochemical Sensor for the Detection of Nicotine. Mater. Adv..

[29] Jerome R., Sundramoorthy A.K. (2020). Preparation of Hexagonal Boron Nitride Doped Graphene Film Modified Sensor for Selective Electrochemical Detection of Nicotine in Tobacco Sample. Anal. Chim. Acta.

[30] Murugan N., Kumar T.H.V., Devi N.R., Sundramoorthy A.K. (2019). A Flower-Structured MoS_2_-Decorated f-MWCNTs/ZnO Hybrid Nanocomposite-Modified Sensor for the Selective Electrochemical Detection of Vitamin C. New J. Chem..

[31] Zribi R., Neri G. (2020). Mo-Based Layered Nanostructures for the Electrochemical Sensing of Biomolecules. Sensors.

[32] Samy O., Zeng S., Birowosuto M.D., El Moutaouakil A. (2021). A Review on MoS_2_ Properties, Synthesis, Sensing Applications and Challenges. Crystals.

[33] Catania F., Marras E., Giorcelli M., Jagdale P., Lavagna L., Tagliaferro A., Bartoli M. (2021). A Review on Recent Advancements of Graphene and Graphene-Related Materials in Biological Applications. Appl. Sci..

[34] Rubio N., Au H., Coulter G.O., Guetaz L., Gebel G., Mattevi C., Shaffer M.S.P. (2021). Effect of Graphene Flake Size on Functionalisation: Quantifying Reaction Extent and Imaging Locus with Single Pt Atom Tags. Chem. Sci..

[35] Lim K.R.G., Shekhirev M., Wyatt B.C., Anasori B., Gogotsi Y., Seh Z.W. (2022). Fundamentals of MXene Synthesis. Nat. Synth..

[36] Rajendran J., Sundramoorthy A.K., Ganapathy D., Atchudan R., Habila M.A., Nallaswamy D. (2022). 2D MXene/Graphene Nanocomposite Preparation and Its Electrochemical Performance towards the Identification of Nicotine Level in Human Saliva. J. Hazard. Mater..

[37] Pang J., Mendes R.G., Bachmatiuk A., Zhao L., Ta H.Q., Gemming T., Liu H., Liu Z., Rummeli M.H. (2019). Applications of 2D MXenes in Energy Conversion and Storage Systems. Chem. Soc. Rev..

[38] Huang W.-X., Li Z.-P., Li D.-D., Hu Z.-H., Wu C., Lv K.-L., Li Q. (2022). Ti_3_C_2_ MXene: Recent Progress in Its Fundamentals, Synthesis, and Applications. Rare Met..

[39] Wang Q., Han N., Shen Z., Li X., Chen Z., Cao Y., Si W., Wang F., Ni B.-J., Thakur V.K. (2022). MXene-Based Electrochemical (Bio) Sensors for Sustainable Applications: Roadmap for Future Advanced Materials. Nano Mater. Sci..

[40] Khazaei M., Mishra A., Venkataramanan N.S., Singh A.K., Yunoki S. (2019). Recent Advances in MXenes: From Fundamentals to Applications. Curr. Opin. Solid State Mater. Sci..

[41] Zhao Q., Yu H., Hu D., Li L.-L., Jin J., Ai M.-J., Wei J., Song K. (2022). Recent Advances in Electrochemical Sensors Based on Palladium Nanoparticles. Chin. J. Anal. Chem..

[42] Chen X., Cai Z., Huang Z., Oyama M., Jiang Y., Chen X. (2013). Ultrafine Palladium Nanoparticles Grown on Graphene Nanosheets for Enhanced Electrochemical Sensing of Hydrogen Peroxide. Electrochim. Acta.

[43] Murugan N., Jerome R., Preethika M., Sundaramurthy A., Sundramoorthy A.K. (2021). 2D-Titanium Carbide (MXene) Based Selective Electrochemical Sensor for Simultaneous Detection of Ascorbic Acid, Dopamine and Uric Acid. J. Mater. Sci. Technol..

[44] Challagulla S., Tarafder K., Ganesan R., Roy S. (2017). Structure Sensitive Photocatalytic Reduction of Nitroarenes over TiO_2_. Sci. Rep..

[45] Hu T., Wang J., Zhang H., Li Z., Hu M., Wang X. (2015). Vibrational Properties of Ti_3_C_2_ and Ti_3_C_2_T_2_ (T = O, F, OH) Monosheets by First-Principles Calculations: A Comparative Study. Phys. Chem. Chem. Phys..

[46] Zhang L., Su W., Huang Y., Li H., Fu L., Song K., Huang X., Yu J., Lin C.-T. (2018). In Situ High-Pressure X-ray Diffraction and Raman Spectroscopy Study of Ti_3_C_2_T_x_ MXene. Nanoscale Res. Lett..

[47] Armano A., Agnello S. (2019). Two-Dimensional Carbon: A Review of Synthesis Methods, and Electronic, Optical, and Vibrational Properties of Single-Layer Graphene. C J. Carbon Res..

[48] Al-Sherbini A.-S., Bakr M., Ghoneim I., Saad M. (2017). Exfoliation of Graphene Sheets via High Energy Wet Milling of Graphite in 2-Ethylhexanol and Kerosene. J. Adv. Res..

[49] Yan P., Zhang R., Jia J., Wu C., Zhou A., Xu J., Zhang X. (2015). Enhanced Supercapacitive Performance of Delaminated Two-Dimensional Titanium Carbide/Carbon Nanotube Composites in Alkaline Electrolyte. J. Power Sources.

[50] Raj S.M.M., Sundramoorthy A.K., Atchudan R., Ganapathy D., Khosla A. (2022). Review—Recent Trends on the Synthesis and Different Characterization Tools for MXenes and Their Emerging Applications. J. Electrochem. Soc..

[51] Applestone D., Manthiram A. (2012). Cu_6_Sn_5_–TiC–C Nanocomposite Alloy Anodes with High Volumetric Capacity for Lithium Ion Batteries. RSC Adv..

[52] Qi B., Di L.-B., Xu W., Zhang X. (2014). Dry Plasma Reduction to Prepare a High Performance Pd/C Catalyst at Atmospheric Pressure for CO Oxidation. J. Mater. Chem. A.

[53] Molaei R., Farhadi K., Forough M., Hajizadeh S. (2018). Green Biological Fabrication and Characterization of Highly Monodisperse Palladium Nanoparticles Using Pistacia Atlantica Fruit Broth. J. Nanostruct..

[54] Mahmood M., Rasheed A., Ayman I., Rasheed T., Munir S., Ajmal S., Agboola P., Warsi M., Shahid M. (2021). Synthesis of Ultrathin MnO 2 Nanowire-Intercalated 2D-MXenes for High-Performance Hybrid Supercapacitors. Energy Fuels.

[55] Magar H.S., Hassan R.Y.A., Mulchandani A. (2021). Electrochemical Impedance Spectroscopy (EIS): Principles, Construction, and Biosensing Applications. Sensors.

[56] Xu A., Weng Y., Zhao R. (2020). Permeability and Equivalent Circuit Model of Ionically Conductive Mortar Using Electrochemical Workstation. Materials.

[57] Mehmeti E., Kilic T., Laur C., Carrara S. (2020). Electrochemical Determination of Nicotine in Smokers’ Sweat. Microchem. J..

[58] Suffredini H.B., Santos M.C., De Souza D., Codognoto L., Homem-de-Mello P., Honório K.M., da Silva A.B.F., Machado S.A.S., Avaca L.A. (2005). Electrochemical Behavior of Nicotine Studied by Voltammetric Techniques at Boron-Doped Diamond Electrodes. Anal. Lett..

[59] Leftheriotis G., Papaefthimiou S., Yianoulis P. (2007). Dependence of the Estimated Diffusion Coefficient of LixWO3 Films on the Scan Rate of Cyclic Voltammetry Experiments. Solid State Ion..

[60] Opitz M., Yue J., Wallauer J., Smarsly B., Roling B. (2015). Mechanisms of Charge Storage in Nanoparticulate TiO_2_ and Li_4_Ti_5_O_12_ Anodes: New Insights from Scan Rate-Dependent Cyclic Voltammetry. Electrochim. Acta.

[61] Abd-Elsabour M., Alsoghier H.M., Alhamzani A.G., Abou-Krisha M.M., Yousef T.A., Assaf H.F. (2022). A Novel Electrochemical Sensor for Detection of Nicotine in Tobacco Products Based on Graphene Oxide Nanosheets Conjugated with (1,2-Naphthoquinone-4-Sulphonic Acid) Modified Glassy Carbon Electrode. Nanomaterials.

[62] Fekry A.M., Azab S.M., Shehata M., Ameer M.A. (2015). A Novel Electrochemical Nicotine Sensor Based on Cerium Nanoparticles with Anionic Surfactant. RSC Adv..

[63] Cavalheiro É.T.G., Brajter-Toth A. (1999). Amperometric Determination of Xanthine and Hypoxanthine at Carbon Electrodes. Effect of Surface Activity and the Instrumental Parameters on the Sensitivity and the Limit of Detection. J. Pharm. Biomed. Anal..

[64] Wu C.-T., Chen P.-Y., Chen J.-G., Suryanarayanan V., Ho K.-C. (2009). Detection of Nicotine Based on Molecularly Imprinted TiO_2_-Modified Electrodes. Anal. Chim. Acta.

[65] Wang S.-J., Liaw H.-W., Tsai Y.-C. (2009). Low Potential Detection of Nicotine at Multiwalled Carbon Nanotube–Alumina-Coated Silica Nanocomposite. Electrochem. Commun..

[66] Xiong H., Zhao Y., Liu P., Zhang X., Wang S. (2010). Electrochemical Properties and the Determination of Nicotine at a Multi-Walled Carbon Nanotubes Modified Glassy Carbon Electrode. Microchim. Acta.

[67] Sims M.J., Rees N.V., Dickinson E.J.F., Compton R.G. (2010). Effects of Thin-Layer Diffusion in the Electrochemical Detection of Nicotine on Basal Plane Pyrolytic Graphite (BPPG) Electrodes Modified with Layers of Multi-Walled Carbon Nanotubes (MWCNT-BPPG). Sens. Actuators B Chem..

[68] Shehata M., Azab S.M., Fekry A.M., Ameer M.A. (2016). Nano-TiO2 Modified Carbon Paste Sensor for Electrochemical Nicotine Detection Using Anionic Surfactant. Biosens. Bioelectron..

[69] Lo T.W.B., Aldous L., Compton R.G. (2012). The Use of Nano-Carbon as an Alternative to Multi-Walled Carbon Nanotubes in Modified Electrodes for Adsorptive Stripping Voltammetry. Sens. Actuators B Chem..

[70] Highton L., Kadara R.O., Jenkinson N., Logan Riehl B., Banks C.E. (2009). Metallic Free Carbon Nanotube Cluster Modified Screen Printed Electrodes for the Sensing of Nicotine in Artificial Saliva. Electroanalysis.

[71] Geto A., Amare M., Tessema M., Admassie S. (2012). Voltammetric Determination of Nicotine at Poly(4-Amino-3-Hydroxynaphthalene Sulfonic Acid)-Modified Glassy Carbon Electrode. Electroanalysis.

[72] Levent A., Yardim Y., Senturk Z. (2009). Voltammetric Behavior of Nicotine at Pencil Graphite Electrode and Its Enhancement Determination in the Presence of Anionic Surfactant. Electrochim. Acta.

[73] Kassa H., Geto A., Admassie S. (2013). Voltammetric Determination of Nicotine in Cigarette Tobacco at Electrochemically Activated Glassy Carbon Electrode. Bull. Chem. Soc. Ethiop..

[74] Papadoyannis I.N., Samanidou V.F., Stefanidou P.G. (2002). Clinical Assay of Nicotine and Its Metabolite, Cotinine, in Body Fluids by HPLC Following Solid Phase Extraction. J. Liq. Chromatogr. Relat. Technol..

