# Supplementary material for: Palladium Hydroxide (Pearlman’s Catalyst) Doped MXene (Ti3C2Tx) Composite Modified Electrode for Selective Detection of Nicotine in Human Sweat
Source: Biosensors (Basel). 2022 Dec 29;13(1):54. doi: 10.3390/bios13010054 (PMC9856038; doi:10.3390/bios13010054)
Supplement: Supplementary file 1 [file biosensors-13-00054-s001.zip › biosensors-2054103-supplementary.pdf]

Palladium Hydroxide (Pearlman's Catalyst) Doped MXene ($Ti_3C_2T_x$) Composite Modified Electrode for Selective Detection of Nicotine in Human Sweat

Vasanth Magesh [1,†], Ashok K. Sundramoorthy [1,*], Dhanraj Ganapathy [1], Raji Atchudan [2,†], Sandeep Arya [3], Razan A. Alshgari [4] and Ahmed Muteb Aljuwayid [4]

[1] Centre for Nano-Biosensors, Department of Prosthodontics, Saveetha Dental College and Hospitals, Saveetha Institute of Medical and Technical Sciences, Poonamallee High Road, Velappanchavadi, Chennai 600077, Tamil Nadu, India
[2] School of Chemical Engineering, Yeungnam University, Gyeongsan 38541, Republic of Korea
[3] Department of Physics, University of Jammu, Jammu 180006, Jammu and Kashmir, India
[4] Department of Chemistry, College of Science, King Saud University, Riyadh 11451, Saudi Arabia
* Correspondence: ashok.sundramoorthy@gmail.com
† These authors contributed equally to this work.

Supporting Figures:

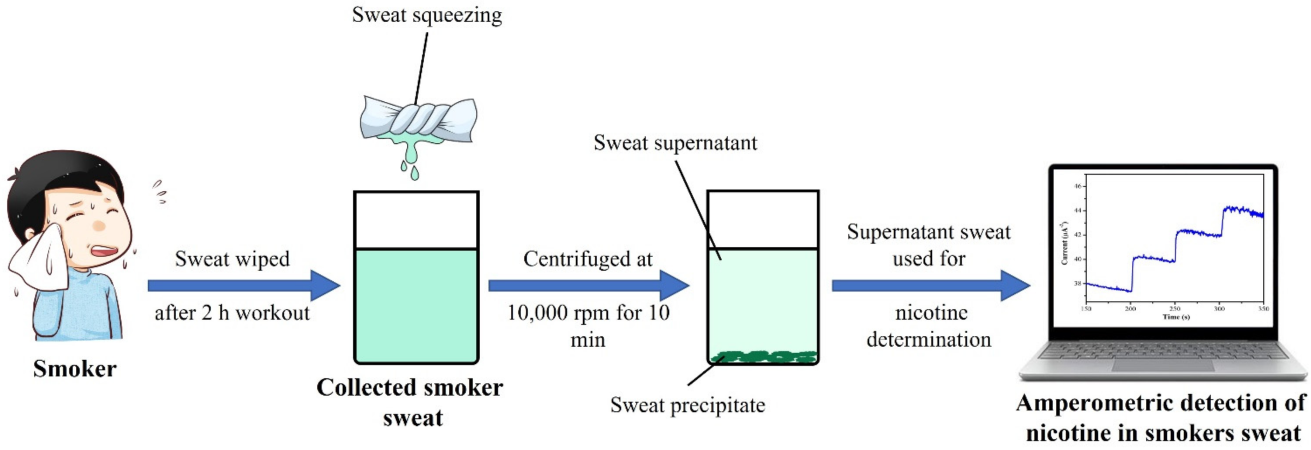

Figure S1. Schematic illustration of sweat sample collection and electrochemical analysis of nicotine.

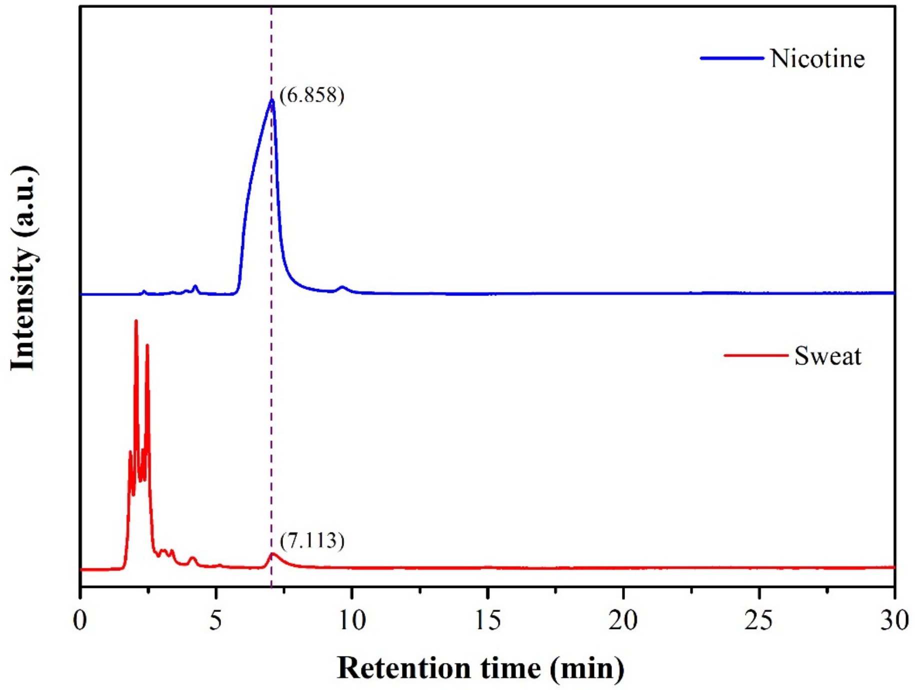

Figure S2. HPLC spectra of standard nicotine (NIC) solution (blue curve) and real sweat (red curve) sample were recorded. This experiment was carried out using a two-phase elution system. The mobile phase A was 0.05M ammonium acetate and the mobile phase B was 100% methanol taken at the ratio of 60:40. It is pumped isocratically at a flow rate of 1.4 ml/min. The intake pressure was 290 kg/cm^2, and the eluent was measured at a wavelength of 262 nm [1]. Initially, 10 μL of known concentration of std. NIC (10 mM) was injected, NIC peak Rt (retention time) was obtained at 6.858. Then, 10 μL of unknown concentration of real sweat was injected, where the NIC peak was obtained at Rt 7.113. The HPLC spectrum revealed that

the retention times for pure NIC and sweat are quite similar. It indicated that sweat sample contains nicotine.

Supporting Reference

1. Papadoyannis, I.N.; Samanidou, V.F.; Stefanidou, P.G. Clinical Assay of Nicotine and Its Metabolite, Cotinine, in Body Fluids by HPLC Following Solid Phase Extraction. *J. Liq. Chromatogr. Relat. Technol.* **2002**, *25*, 2315–2335, doi:10.1081/JLC-120014006.
